# Anterior Lumbar Interbody Implants: Importance of the Interdevice Distance

**DOI:** 10.4061/2011/176497

**Published:** 2011-03-10

**Authors:** Brian R. Subach, Anne G. Copay, Marcus M. Martin, Thomas C. Schuler

**Affiliations:** ^1^The Virginia Spine Institute, Reston, VA 20190, USA; ^2^The Spinal Research Foundation, 1831 Wiehle Avenue, Suite 200, Reston, VA 20190, USA

## Abstract

*Object*. The implantation of interbody fusion cages allows for the restoration of disc height and the enlargement of the neuroforaminal space. The purpose of this study was to compare the extent of subsidence occurring after conventional cage placement compared to a novel wider cage placement technique. *Methods*. This study is a retrospective evaluation of radiographs of patients who underwent stand-alone single level anterior lumbar interbody fusion with lordotic titanium cages and rhBMP-2. Fifty-three patients were evaluated: 39 patients had wide cage placement (6 mm interdevice distance) and 14 had narrow cage placement (2 mm interdevice distance). Anterior and posterior intervertebral disc space heights were measured post-operatively and at follow-up imaging. *Results*. The decrease in anterior intervertebral disc space height was 2.05 mm versus 3.92 mm (*P* < .005) and 1.08 mm versus 3.06 mm in posterior disc space height for the wide cage placement and the narrow cage placement respectively. The proportion of patients with subsidence greater than 2 mm was 41.0% in the wide cage patients and 85.7% for the narrow cage patients (*P* < .005). *Conclusions*. The wider cage placement significantly reduced the amount of subsidence while allowing for a greater exposed surface area for interbody fusion.

## 1. Introduction

The use of cages for interbody fusion has rapidly increased in recent years. The advantages of an interbody cage include the restoration of disc height and the enlargement of the neuroforaminal space. Those advantages are essentially nullified by subsidence of the cage into the adjoining vertebrae. Clinical factors such as the quality of the bone tissue and the weight of the patient, have been found to influence subsidence [1]. Cage-related factors also determine the incidence of subsidence, including cage design (shape and surface contact area) and cage position within the disc space.

Studies have shown that a larger area of contact between endplate and cage produced a lower stress distribution pattern [2] and that bone grafts covering more than 30% of the endplate area were able to carry significantly greater loads [3]. There is overwhelming evidence that the characteristics of the endplate are not uniform throughout its surface, a fact that should impact the positioning of an interbody device. A biomechanical study by Grant et al. [4] demonstrated that the posterolateral region of the lumbar endplate (ring apophysis) is twice as strong as the central area. Similarly, Lowe et al. [5] found the highest maximum load to failure to be in the posterolateral region of the endplate just anterior to the pedicle. Their study also showed that the central portion of the endplate had a thin cortex and provided little resistance to a compressive load. Sohn et al. [6] reported that a more lateral cage placement produced a distribution of strains closer to physiological than a ventral cage placement. Titanium cages placed posterolaterally had a 20% higher failure load than centrally placed cages, even though the difference was not significant [7]. Those biomechanical studies were corroborated by a finite-element analysis concluding that cages should rest on the strong peripheral part of the endplate [8]. 

Clinical studies have reported a wide range of subsidence occurrence: from 3% and 4.5% for BAK standard cages [9, 10], to 15% for BAK Proximity [9], and to 76.7% for rectangular cages [11]. Clearly, efforts should be made to optimize cage placement in order to reduce cage subsidence. However, a posterolateral placement of the interbody device carries other risks. McAfee et al. [12] found that a cage placement too lateral may result in a far lateral disc herniation compromising the nerve root. The most common reason for the unintentional lateral cage placement was failure to identify the anatomic midline. Taylor et al. [13] systematically varied the cage placement and observed its impact on foraminal violation. A BAK cage placed at the midline did not cause any foraminal violation; placed 10% laterally (5.29 mm from the midline), it caused foraminal violation in 17% of the cases; placed 20% laterally (9.78 mm from the midline) it caused foraminal violation in 50% of the cases.

In a desire to improve upon the technique of anterior lumbar interbody fusion (ALIF), we began using a slightly wider placement of the titanium fusion cages than used in current techniques. Such positioning allows for better approximation of the cages with the greater load-bearing capacity of the vertebral ring apophysis. In addition, the wider placement of ALIF cages increases the available protected surface area for fusion by increasing the interdevice area between the cages themselves. With a narrower cage placement, excess bone graft is placed in any available space lateral to the implant in an attempt to augment the fusion. Such areas are poorly decorticated and poorly vascularized sites for arthrodesis.

The purpose of the current study is to compare the rate of subsidence following the standard and wide placement of titanium cages. In standard cage placement, the cages are spaced 2 mm apart (1 mm away from the midline). In the wide cage placement used in this study, the cages are spaced 6 mm apart (3 mm away from the midline). This study's wide placement is still narrower than Taylor's 10% lateral placement. It is hypothesized that the 3 mm cage placement will reduce the incidence of subsidence.

## 2. Materials and Methods

### 2.1. Surgical Procedures

**Figure 1:**
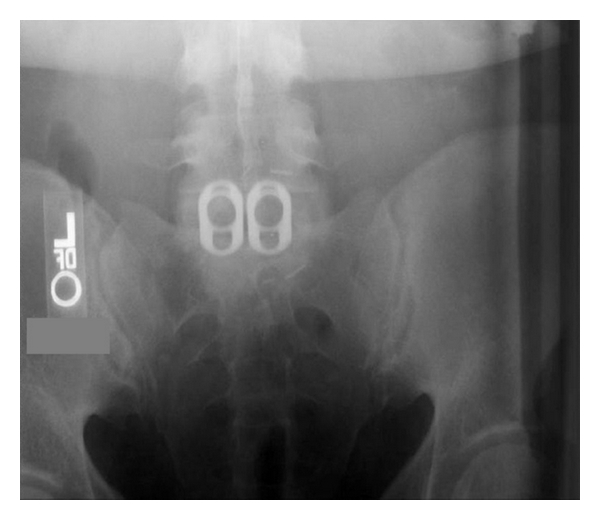
Standard narrow cage placement at L5-S1 level.

The standard anterior lumbar interbody fusion (ALIF) technique to place the LT-CAGE Lumbar Tapered Fusion Device (Medtronic Sofamor Danek, Memphis, TN) into the L4-L5 and L5-S1 levels relies on a retroperitoneal approach to the spine. The standard insertion technique involved a block discectomy and sequential distraction of the target disc space under fluoroscopic guidance followed by placement of a double-lumen guide tube. Reaming and cage placement through the guide allowed for placement of the cages with a 2-mm interdevice distance (Figure 1). After adequate positioning was obtained, bone morphogenetic protein (rh-BMP-2) was placed in the cages based upon suggested dosing by the manufacturer. 

**Figure 2:**
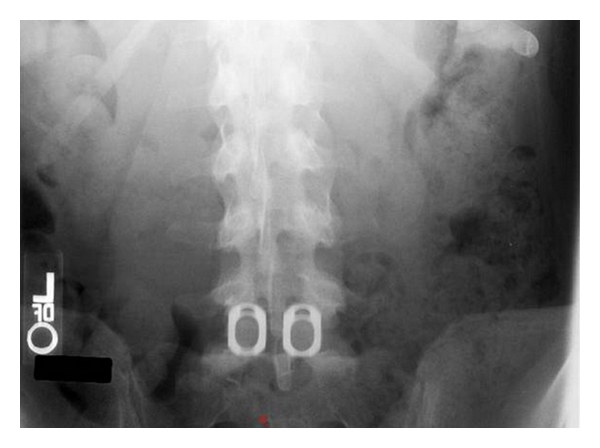
Wide Cage placement at L5-S1 level.

The wide insertion technique followed the steps outlined for the standard technique just described; however, after the discectomy and distraction of the target disc space, a custom double-lumen guide tube was used for reaming and cage placement, which is carried out in the standard manner through both tubes. The standard guide tube leaves a 2-mm space between the devices while the custom tube creates a 6-mm space and a more lateral placement of the cages in the disc space. The tubular guide was removed and the 6-mm interdevice area of end plate was decorticated with a small curette, drill, or osteotome (Figure 2). After adequate positioning was verified by fluoroscopy, rh-BMP-2 was placed within the cages and in the interdevice area.

### 2.2. Sample

A search of the electronic patient database identified patients who underwent stand-alone ALIF at L4-L5 or L5-S1 between 2002 and 2006. If postoperative and follow-up X-rays were available in the database, those patients were included in the sample. A total of 47 patients had adequate X-rays: 12 patients had a narrow cage placement and 35 patients a wide cage placement. The patients had an average age of 44.0 years and an average BMI of 26.8 kg/m^2^. This sample of patients was 51.1% female and 68.1% nonsmokers.

### 2.3. Assessment of Subsidence

**Figure 3:**
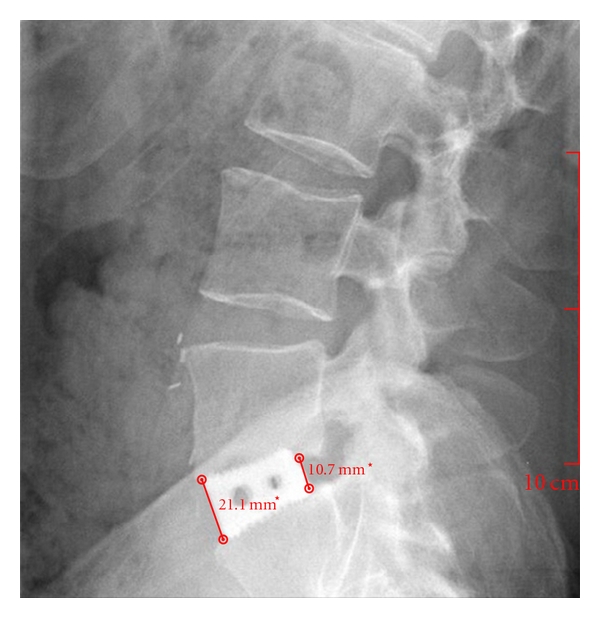
Measurement of digital X-ray.

**Figure 4:**
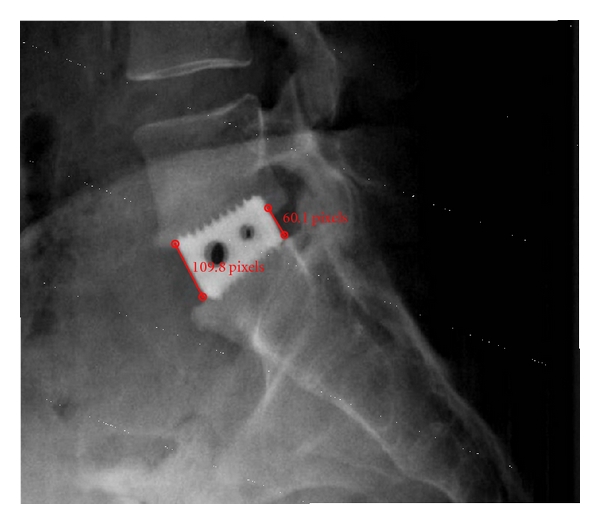
Measurement of scanned analog X-ray.

The height of the intervertebral disc space was measured using lateral X-rays taken at the postsurgery visit (10 to 15 days postsurgery) and at a follow-up visit (3 to 51 months postsurgery). Radiographs were measured by the spinal surgeon (BRS) using an automated image analysis software (Echoes, Medstrat, Downers Grove, IL). Of the 106 X-rays, 56 were originally digital images and 50 were analog films. The measurements of the digital images were in real units (mm) (Figure 3). The 50 analog films X-rays were scanned into the digital X-rays software. The measurements of the scanned X-rays were in pixels. The cage was also measured on the analog film and the known dimension of the interbody cage was used as a correction factor to transform the pixels into mm (Figure 4). Measurements were recorded for both the anterior and posterior regions of the intervertebral disc space by measuring from superior endplate to inferior endplate.

In this study, subsidence is defined as the reduction in disk space from postoperative to follow-up visit. An alternative definition of subsidence as a decrease in disc space greater than 2 mm [11] was used. If either the anterior or posterior disc space had decreased by more than 2 mm, patients were classified as having subsidence.

### 2.4. Data Analyses

Statistical analyses were carried out with SPSS (version 15.0, SPSS Inc., Chicago, IL). Patient characteristics and subsidence were compared between the two groups using *t*-tests for independent samples for numerical data and using chi-square for categorical data. Pearson correlation coefficients were calculated to assess the relationship between patient characteristics and subsidence.

## 3. Results

Baseline characteristics were not different between the two groups of patients (Table 1).

Subsidence was significantly greater in the narrow cage group than in the wide cage group (Table 2).

Subsidence at the anterior region and subsidence at posterior region of the intervertebral disc were significantly related (Pearson *r* = .47, *P* < .0001), while BMI, body weight, smoking status, and length of time to follow-up were not significantly related to the amount of subsidence.

When subsidence was defined as a loss of disc space greater than 2 mm [11], subsidence occurred in 83.3% of the narrow cage patients as opposed to 42.9% of the wide cage patients (*P* < .05).

## 4. Discussion

The wide placement of the cages markedly reduced the loss of intervertebral space. The loss of intervertebral space was nearly two times (anterior region) and three times (posterior region) less with the wide cage placement. When subsidence was defined as a loss of disc space greater than 2 mm, subsidence occurred in a larger proportion of the narrow cage patients than the wide cage patients as well.

Amounts and rates of subsidence have been reported across a variety of surgical techniques and devices (including bone grafts and instrumented fusions) and calculated with various operationalizations of subsidence: a greater than 10% loss of height [14], greater than 3 mm [15], or greater than 2 mm [9]. The patients in our sample had a 1-level noninstrumented ALIF with metallic cages. In a comparable sample, 56 patients underwent 1-, 2-, or 3-level noninstrumented ALIF with BAK proximity cages [16]. The average subsidence was 1.97 mm in the anterior region and 0.82 mm in the posterior region. Choi and Sung [11] found that 76.6% of their patients developed subsidence (greater than 2 mm) following a single-level noninstrumented ALIF with rectangular cages. Similarly, Beutler and Peppelman [9] reported a 10% rate of subsidence (greater than 2 mm) after 1- or 2-level ALIF with BAK cages.

The wide range of time elapsed between the postoperative and follow-up assessment is a limitation of this study. The time to follow-up assessment ranged from 3 to 51 months with an average of 19.3 months and a median of 13.3 months. However, the length of time was unrelated to the amount of subsidence. This might be due to the fact that subsidence is likely to occur fairly early after surgery. Choi and Sung [11] reported the onset of subsidence between 0.25 and 8 months, with a median time of 2.75 months. Kumar et al. [17] noted that subsidence occurred within 15 days then minimally increased up to 6 months.

More lateral cage placement carries the risk of foraminal violations, as demonstrated by Taylor et al. [13]. We did not observe any case of foraminal violation in our sample with a cage placement of 3 mm away from the midline. 

While the wider placement of the lordotic cages decreases subsidence, it does not avoid the “plowing” of the cages into the vertebral body at the time of placement. Plowing occurs with both a wide and narrow insertion technique. Although the distraction opens up the disc space, the constraint of the facet joints tends to opens the disc space more anteriorly than posteriorly. The cylindrical reamer always reams into the inferior aspect of L5 and allows the cage to “plow” into the vertebral body. Since the L5-S1 level is the most lordotic, plowing is most commonly seen at this level. The parallel endplates at other levels are distracted in parallel, avoiding the reaming into the inferior endplate of the superior vertebral body.

In general, we believe that the wider spacing should be used in all patients if anatomically possible. If vascular anatomy impairs a centered exposure of the disc space, it is preferable to use narrow cages. Otherwise, we attempt the wide construct in all cases. It is important to remember that wide 14-mm cages restore the same degree of disc space height as narrow 16-mm cages.

## 5. Conclusion

Compared with the results from the standard procedure, the novel technique of device placement described here has dramatically decreased the incidence of subsidence in our patient population. The decreased rate of subsidence theoretically and practically reduces the incidence of foraminal stenosis, pseudarthrosis, kyphosis, and the need for revision surgery.

Furthermore, the interdevice area (exposed surface for interbody fusion) with the novel technique was reproducibly increased threefold over the standard technique. The mean surface area between the cages was increased by 80 mm^2^ for 20-mm length cages, 92 mm^2^ for 23-mm length cages, and 104 mm^2^ for 26-mm length cages. Interdevice volume depends on the cage height while area depends on the interdevice distance. The increased surface area between cages implies increased surface area available for fusion on both the superior and inferior end plates. On the imaging studies, the cages were clearly seated more laterally with regard to the vertebral ring apophysis with clearly visible intervening bone surface available for fusion.

## Figures and Tables

**Table 1:** Patient characteristics.

	Wide cage (SD)	Narrow cage (SD)
Average age (years)	43.2 (8.6)	46.1 (6.2)
Average BMI (kg/m^2^)	26.1 (4.9)	28.9 (4.7)
Average number of months of followup	18.4 (12.8)	21.9 (15.0)
% Female gender	50%	51%
% Nonsmokers	71.4%	58.3%

**Table 2:** Subsidence: mean (mm) and standard deviation.

	Wide cage	Narrow cage
Anterior	2.16 (2.35)	3.50* (1.67)
Posterior	1.25 (1.16)	3.33* (1.73)

*P < .05.
